# ‘She taught me’: factors consumers find important in nurse practitioner and pharmacist prescriber services

**DOI:** 10.1186/s12960-021-00587-y

**Published:** 2021-03-26

**Authors:** Tara N. Officer, Jackie Cumming, Karen McBride-Henry

**Affiliations:** 1 Health Services Research Centre, Victoria University of Wellington, Pipitea Campus, PO Box 600, Wellington, New Zealand; 2 School of Nursing, Midwifery, and Health Practice, Victoria University of Wellington, Newtown Campus, PO Box 7625, Wellington, New Zealand

**Keywords:** Nurse practitioner, Pharmacist prescriber, Patient-centred care, Realist evaluation, Non-medical prescribing, Consumer/patient perspective

## Abstract

**Background:**

Advanced practitioner services, such as those nurse practitioners and pharmacist prescribers provide, are an opportunity to improve health care delivery. In New Zealand, these practitioners remain underutilised, despite research suggesting they offer safe and effective care, and considerable international literature recording patient satisfaction with these roles. This study aimed to explore factors underlying consumer satisfaction with primary health care nurse practitioner and pharmacist prescriber services.

**Methods:**

As part of a larger realist evaluation, 21 individuals receiving advanced practitioner services participated in semi-structured interviews. These interviews were transcribed and coded against context–mechanism–outcome configurations tested and refined throughout the research.

**Results:**

Study findings emphasise the importance of consumer confidence in the provider as a mechanism for establishing advanced practitioner roles. Underlying this confidence is a recognition that these practitioners work in a more accessible manner, engage at the individual’s ‘level’, and operate with passion.

**Conclusions:**

This research offers learnings to re-engineer service delivery within primary health care to make best use of the entire health care team by including consumers in the design and introduction of new roles.

## Background

Recent health workforce planning and policy initiatives aim to meet expected increases in health service demand through increasing workforce numbers and skills. A challenge in the current primary health care climate of general practitioner (GP) shortages [1, 2] and constrained spending is how best to introduce new workforce roles to enable patient-centred care [3] and continuity of service provision. This is particularly important in New Zealand’s culturally diverse population, where the indigenous Māori population have generally poorer health access and outcomes across their life course [4]. The creation of nurse practitioner (NP) and pharmacist prescriber (PP) roles intended to improve patient health service access and make better use of workforce skills [5–7]. Curiously, this workforce has remained largely underutilised. Many nurses have completed prerequisite Masters level training, but have not registered as NPs [8]. Similarly, since being gazetted in 2013, there are only 20 currently registered and practising PP [9]. Growth in numbers for these professions has fallen below early projections [10, 11].

Globally, there is increasing acceptance and interest in advanced practitioner roles and their contribution to improved health outcomes. Studies have suggested that NP and PP services provide improved health service accessibility [12–16], patient satisfaction, good patient experience, and equivalent quality of care as traditional health care providers [17–25]. Several randomised control trials of NP care, compared to GP care, reinforce arguments for enhanced or comparable patient satisfaction, with improved individual knowledge following NP consultation [19, 20, 26, 27]. More recently, Jebara and colleagues’ (2018) systematic review of stakeholder experiences with pharmacist prescribing suggests that consumers with experience of PP roles are satisfied with their services, but that often satisfaction is limited to treatment within clearly defined parameters [28]. Arguably, findings are often compromised by an inability to attribute satisfaction definitively to activities advanced practitioners perform.

Few studies have explored factors underlying consumer satisfaction with advanced practitioner services. No studies have examined these views on PP-provided services in New Zealand. As part of understanding the development of NP and PP roles in New Zealand primary health care, this article lays out consumer (patients or other individuals accessing NP/PP services) perspectives and identifies what works for them and why it does. In so doing, this article informs discussion of consumer experience with health care delivered by advanced practitioners.

## Method

Pawson and Tilley’s (1997) [29] realist evaluation methodology, a theory-based approach, sits well with an evaluation of programmes sharing a family resemblance [30], such as the development of NP/PP roles. A central tenet of this approach is that context influences whether mechanisms (changes in the resources and reasoning) are triggered (and to what extent they are triggered) and lead to outcomes [29]. Hence, the focus of evaluation shifts from whether outcomes occur to testing and refining theories about how they (fail to) occur [29].

Given the lack of extant theory covering the development of NP and PP roles in New Zealand, a realist evaluation approach was applied to this project. Realist evaluations work under the principle that stakeholders contribute diverse perspectives because of their roles in a programme. Advanced practitioners operate in a complex health system environment, where there are many in and outflows, and multiple parties working in differing contexts, influencing the successful operation of these roles. These parties are not all-knowing, instead, their knowledge of how roles develop differs based on their experience; they consequently provide different perspectives and will respond differently to theories proposed to them [31]. In this research, NP/PP development is subject to the reasoning, choices, and resources of those using, and affecting change in, these roles. Consequently, because of their ability to contribute judgements to forming configurations of context, mechanism, and outcome [29, 32], as part of the wider research project [33], we gathered information from parties across the spectrum of opinion (see “Data collection” section, below), including consumers, to form a picture of advanced practitioner role development. These individuals were able to contribute to answering the principal question of realist evaluation, which is ‘what works, for whom, in what circumstances, to what extent, and why?’ [34]. For this research, the emphasis on mechanisms (what works and why) was vital given the different worldviews of parties involved in creating and implementing these roles. Consumer views, articulated in this article, offer one perspective on NP/PP roles and the potential contexts and mechanisms facilitating their success.

### Data collection

**Fig. 1:**
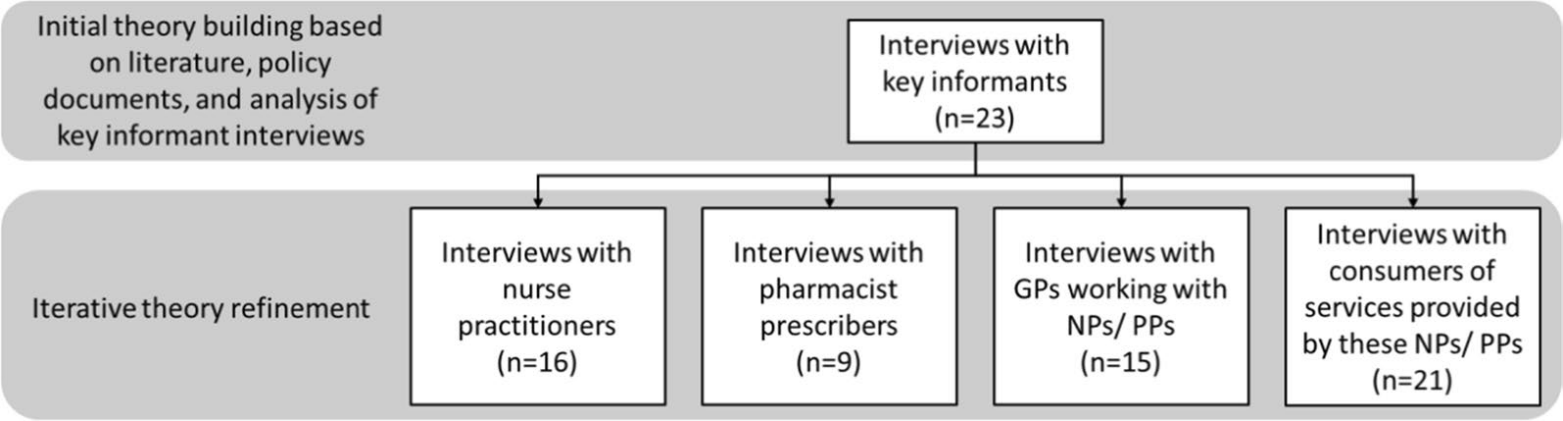
Overall data collection

The Victoria University of Wellington Human Ethics Committee approved this project in December 2015 (#22388). The present article outlines findings of interviews conducted with consumers of PP (*n* = 9) and NP services (*n* = 12). These interviews occurred following interviews with key training, policy, and advocacy informants, and concomitant with NP, PP, and GP interviews (Fig. 1). In total, 84 individuals participated in the full study; theories developed through literature were tested during key informant interviews and subsequently refined and re-tested during health professional and consumer interviews.

Advanced practitioners recruited consumers for this research based on set criteria (Table 1). Where patients were under 18 years, or otherwise unable to consent, then a parent or other individual hiring advanced practitioner services for the patient was invited to participate in this research.

**Table 1:** Criteria for participant selection

Inclusion criteria
Received services from their NP or PP at least once in the preceding year
Currently receiving services from their NP or PP
Not acutely ill or facing extenuating family circumstances
Over 18 years old
Able to provide informed consent

NPs/PPs generated a blinded list of individuals meeting the above criteria from their daily appointment register. Each day, the NP/PP randomly selected a consumer from this list for four consecutive working days. Where the advanced practitioner treated small populations, individuals unable to consent, or had only recently commenced employment, then fewer people were approached. Participating NPs/PPs explained the research to the sampled consumers and then supplied them with a letter inviting them to contact the primary investigator, an information sheet describing the research, and a consent form. When parties made initial contact, the primary investigator confirmed their understanding of the research and agreed an interview time. Interview numbers were set by the number of people each practitioner recruited. Twenty-eight people returned consent forms; two did not provide contact details and five returned forms after data collection ceased.

Participants supplied written informed consent before participating in this research. They were advised they could pause or stop their interview at any time, bring additional support to their interview, and not have their interview recorded. No one took up these options. The primary investigator conducted interviews in mutually agreed locations; these interviews concluded when participants felt they had nothing more to add and lasted between 10 and 45 min. All participants were advised that the primary investigator was a pharmacist.

An interview schedule (Table 2) was created to facilitate open-ended questioning; interviews adhered to the principles of realist teacher–learner cycles [32, 35]. This involves teaching interviewees about hypothesised theories so that they could respond in relevant ways to the proposed theories and discuss their appropriateness and relevance [31, 35]. Interviews using a realist approach are as a form of idea exchange so that interviewees iteratively confirmed, disproved, or refined theories [29]. Participants had the opportunity to elaborate on their experiences, in turn, these experiences illuminated the contexts, mechanisms, and outcomes influencing the development of NP/PP roles from their perspectives. As consumer interviews were conducted following interviews with key informants (and their analysis), and concurrently with health professional interviews; theories hypothesised during earlier interviews were posed to interviewees and refined in these interviews.^1^ Participants could request copies of their interview transcript and a summary of their interview. This provided a second opportunity for participants to review hypothesised theories (in the summary documents) and refine their thinking. It also facilitated the researcher’s early consideration of possible theories and, consequently, provided a second opportunity for teacher–learner engagement when participants reviewed and commented on these summaries. A full breakdown of how the teacher–learner cycle was applied is also available [33].

**Table 2:** Interview schedule

Key questions	Prompts
What is the role of NPs/PPs in your primary health care practice?	Can you please provide some examples of their role in your care?
	What reasons do you have for using NP/PP services over other available care providers?
How does their role differ from what you expected?	Positive factors affecting the consultation process
	Negative factors affecting the consultation process
	How do these factors affect consultations?
What impact does your NP/PP have on your treatment?	What do you most value about NP/PP consultations?
	How does this differ from other consultations you have had?
	How does the NP/PP change your access to care?
Is there anything else that you would like to add?	

### Data analysis

**Fig. 2:**
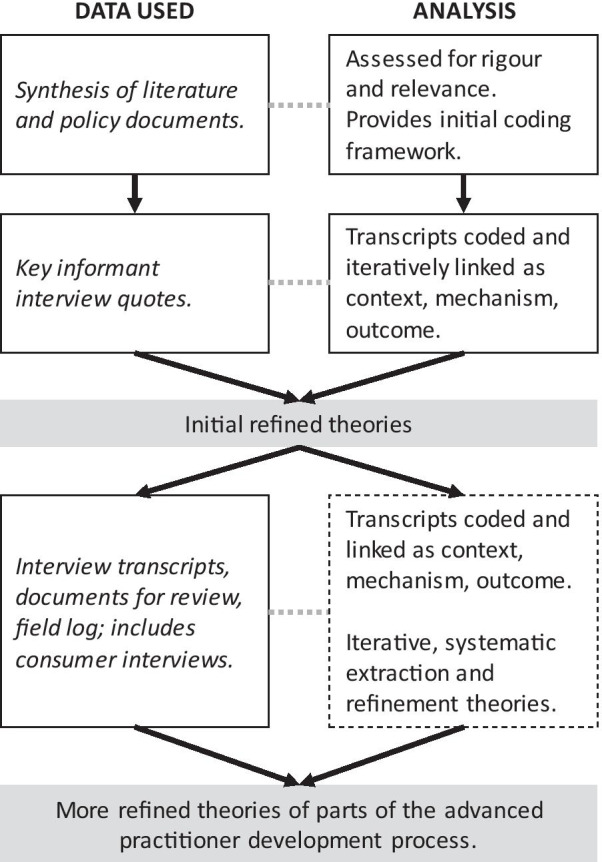
Data analysis

Interviews were recorded and transcribed by a third party working under a confidentiality agreement. The primary investigator checked these transcribed interviews. An initial synthesis of literature on NP/PP primary health care role development [33] acted as a basis on which to formulate a priori theories to test and refine during data collection and analysis. Following a realist evaluation approach, the primary coding structure during data analysis are configurations of context, mechanism, and outcome [36, 37] that lead to the generation of more honed theories. Refinement occurred iteratively and systematically as the influence of context on specific mechanisms became apparent across interviews [38]. Transcripts were coded using NVivo 11 Pro (QSR International) against theories tested during key informant interviews. Analysis continued concurrent with data collection, followed realist evaluation analysis processes [29, 35, 38], and was informed by earlier realist research on the role of the nurse practitioner [39–42], as laid out in Fig. 2.

## Results

Research participants represented a range of ages, genders, socioeconomic statuses, ethnicities, localities, and medical needs. They had varied experience with advanced practitioners, some having enrolled in their general practice within the past year, others having intergenerational relationships with the practice/advanced practitioner. Participants were patients of advanced practitioners (*n* = 18) or were parents, guardians, or another individual hiring NP/PP services (*n* = 3). Advanced practitioners all had extensive experience in their professional fields operating across a range of practice environments. All advanced practitioners met the New Zealand legislative requirements for registration in their respective professional scopes of practice.

Participants described confidence in their advanced practitioner and in their own ability to manage their health as the mechanism leading to satisfaction with the services of their advanced practitioner (Table 3). Three main contexts triggered this mechanism. Interviewees recognised that these providers were patient-centred in their care, participants reported feeling known as individuals. They noted that advanced practitioners discussed treatments, making consumers part of the team. Participants also recognised their advanced practitioner’s passion for their roles.

**Table 3:** Mechanism and contexts generated from participant interviews

Mechanism	Confidence		
Contexts	Being known	Doctors tell, advanced practitioners discuss	Provider passion

### Confidence

In all interviews, the mechanism of confidence was explored. In one case, this confidence came from knowing that PPs were health professionals and were “*very much the same*” (P5) as doctors. More commonly, participants advised that they felt their conditions improved because of their advanced practitioner’s intervention. Consumers noted that they felt part of the team when receiving advanced practitioner services, where they recognised and trusted their advanced practitioner’s skills, and felt the practitioner listened to them. One interviewee described how this confidence changed how they sought health care services:

I wouldn’t send my kids to the doctor with my husband. But I have sent my kids to [the NP] with my husband… I trust the care that she will discuss with him…He doesn’t always get the kind of results I get out of a health consult, but I think she’s very aware of that… She does consult with him, she does give him information in a way that he can consider it. (P20)

Many participants had long-term relationships with providers. Others had been referred to their advanced practitioner by members of the health care team or had first visited their NP/PP as an alternative to their GP. Interviewees described their confidence in providers as occurring irrespective of their knowledge of their advanced practitioner’s skills:

I haven't really noticed any change because to me she has always been the same, although her knowledge… changed because of her schooling up… It's not something you see as a patient. You just know that she knows. You’ve got that confidence that she knows. (P3)

In several cases, participants compared the confidence they had in their advanced practitioner to confidence in other providers. Answers varied from an absolute refusal to seek support if their advanced practitioner was absent or their contract ended, to a feeling that medical practitioners, particularly locum doctors, did not build relationships:

When you go to the doctor, it’s not the same. You’re just in there and they want to hurry up and get rid of you… See, they don’t really care about me…I’m just a bloody number… We’re just dollar signs when we go to the doctors. (P6)

Participants described differences they perceived between their NP/PP and registered nurse or pharmacist, respectively. One participant attributed this difference in confidence to a feeling that their NP operated with greater autonomy than a registered nurse:

I probably wouldn’t have… [taken my son for education] with either just a registered nurse or a doctor… I would feel like they thought I didn’t know…, whereas… that was a role she would pick up and lead really well. I don’t know that I would have had necessarily the confidence in some of the registered nurses because… they’re not working autonomously in the same way. (P20)

Where consumers had unmanaged long-term conditions or needed close monitoring, they often then received PP services. These interviewees explained changes in their usual feelings of frustration because of their PP’s intervention:

It’s given me confidence in myself that if… things aren’t going right, I can turn to somebody who I think cares and knows what to do. Whereas I'll just plug in more insulin and get nowhere… she’s organised it all. (P1)

In turn, this feeling of confidence, both in self and in the provider, was framed around discussions that NPs/PPs operate in a more personalised manner than other practitioners.

### Being known

Participants spoke of the availability of NPs/PPs to deliver services in locations separate from general practice, for example, on marae (a Māori meeting place) or rural hubs. They considered advanced practitioners to have more time to spend managing health care needs and often noted shorter appointment wait times. Interviewees described their advanced practitioner as part of their journey to good health, a return to personalised care, and openness to the whole person and their family, rather than a sick patient:

She is so proactive, and you get that old-fashioned personal care again. She doesn’t start typing the script as you walk in. She doesn’t assume that because you're big you need a lecture on weight and then just toss whatever else is wrong… You get to have that faith and trust in her… She gets to know you… that you're not just coming in because you’ve got nothing better to do with your day. (P16)

Interviewees described services from their PP in the same manner:

Just because you have type 2 diabetes doesn’t mean that we all should be on two tablets… because everybody’s body is different, and that’s what she focuses on, that we’re all different. You might need something completely different… everybody’s body works differently. That’s what she’s taught me as well. (P4)

Participants advised that their advanced practitioners understood their needs holistically. They saw this approach to care as patient-centred, accessible, and reflecting individual needs. This led consumers to feel confident that they ‘owned’ and were involved in their own health care.

### Doctors tell, advanced practitioners discuss

Participants commented that advanced practitioners influenced confidence in the care being delivered by working to improve consumer understanding of their care. This understanding occurred because of increased time with NPs/PPs, the language advanced practitioners used, and NPs/PPs working to ensure consumers understood their disease states. The following narrative offers a comparison between GP and NP services:

He [the GP] knows his stuff… and that’s what I like about him… He’s just doing his job and that’s it. Whereas that’s what makes me comfortable with her [the NP], because she kind of is like well “how’s your day going”… It’s a lot more personal… and that’s what I like. It’s explained in the language that we understand… She makes sure that you understand everything before you leave. (P11)

Participants further emphasised the role of advanced practitioners in education and health literacy. They described ‘holistic’ models of care that surfaced underlying health problems and facilitated the use of rongoā (traditional Māori medicine) or other forms of service delivery:

If she goes to the marae, [Māori people] will come. They won’t come in here, into the doctor… He’s up… and we don’t go to him. And also, I think they allow the Māori medicine side to also work… If they went to the doctor, he might say no, this is what you have to do… Where she will listen and work both sides for them. (P18)

Participants discussed being able to communicate with their advanced practitioners, for example, PPs were often available via text message, phone, or email. They emphasised the ‘constant’ that advanced practitioners offered patients and how this influenced care continuity:

So many GPs now you’ve got to a practice and you can never get that constant ongoing care, whereas having… my nurse practitioner, it's like having that constant person that knows you every visit. It's that continuity of care. (P16)

Similarly, interviewees commented that doctors will “tell”, and advanced practitioners “discuss”:

Doctors, they tell you things… But she can sit there just talking for 10 minutes, see how you are, how you’re feeling. You could be there 20 minutes talking to her, it’s not a problem. I find her more important to me. I know more of what’s going on talking with her than with the doctor… He’s got an appointment every 10 minutes, so he’s got to shove you in, shove you through. (P1)

Participants commonly spoke of receiving ‘conveyor belt’ services from GPs, or of feeling that their nurse or GP lacked time to follow-up their care needs. One individual described the difference between receiving specialist care and care from their NP:

The difference… between the specialist in the hospital is, they’re flat out and might be seeing 50 people a day then get home. Whereas with [my NP], she might be seeing 50 people a day but when I’m there I feel like I’m the only one there that’s important at that point in time. (P10)

### Provider passion

Participants described their NP/PP as passionate in their roles as health care providers. It was common for participants to see their advanced practitioner as “*magic*” (P1) and fulfilling their roles as health providers because of a commitment and desire to help others. Many interviewees saw their providers as “*on a mission to do great things*” (P21) for them. They subsequently felt that services were financially worthwhile. In many cases, participants emphasised that by having advanced practitioners working in practices and locations where the NP/PP and their whānau (families), had been based for years, they then fit the practice and grew alongside their patients:

This is her whānau, so she’s more than happy to be here… She’s got roots here, whānau… She fits perfectly into this place. (P6)

Recognition of NP/PP commitment improved consumer confidence in themselves and in the provider.

### Articulating the refined programme theory

**Fig. 3:**
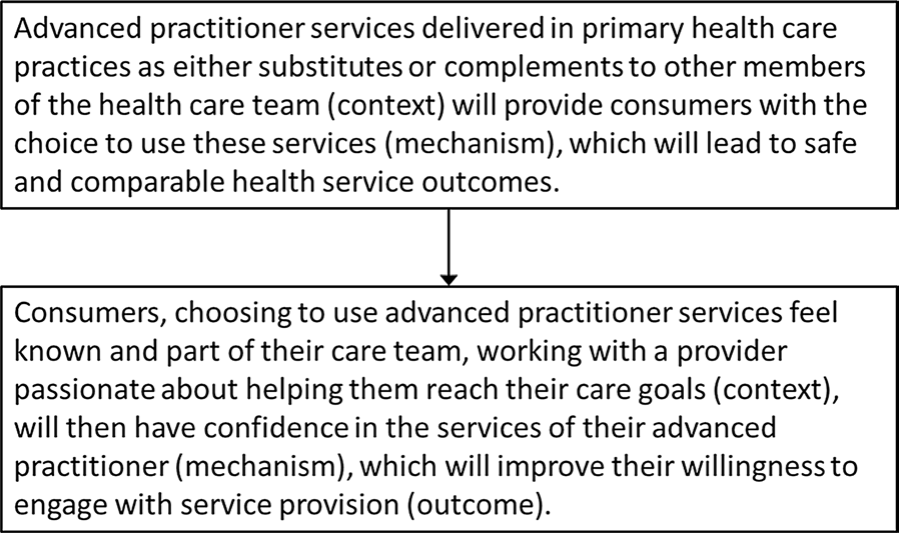
Theory refinement

Acknowledging that this article lays out findings from only one part of the overall research project, applying a realist evaluation methodology to this work presents an opportunity to move iteratively from an initial theory explaining what works for consumers about advanced practitioner services to more refined theory (Fig. 3). In turn, this builds into theories explaining the overall development of NP and PP roles but also invites further research into consumer responsiveness to these roles.

## Discussion

Participants describe receiving NP/PP services as improving their confidence (a mechanism) in their own ability to manage health conditions and in their health care provider. Contexts triggering this included a recognition that advanced practitioners discuss health conditions on the consumer’s ‘level’. Interviewees advised that NPs/PPs know them personally and deliver care in a way that incorporates the individual into the care team. Services delivered by these providers were individualised and empowered interviewees to understand their own conditions. Such findings are supported by earlier literature [18–24, 43], and the myriad of existing frameworks and discussions on key aspects of patient-centred care [3, 44].

Appropriate management of population health needs in primary health care reduces requirements for specialist care. However, in New Zealand and globally, primary health care workforce recruitment is challenging. These challenges, combined with increasing patient requirements, are likely to affect timely access to care [1, 2]. Similarly, known inequities in indigenous Māori health outcomes [4] mean there is a need to change how services are developing. While simply increasing the range of health providers may not improve coordination of care [45], there is scope to improve how we employ health professionals. Ensuring that general practice roles are well-demarcated, clearly defined, and integrated is critical to meeting population needs, so that interplay between various disciplines is strengthened [46]. To this end, consumers should be educated around the distinct roles health care providers perform.

There is growing recognition of the value of consumer involvement in the implementation, delivery, and evaluation of health service initiatives [47, 48]. Despite research suggesting patients are satisfied with advanced practitioner services, often, introduction of these roles has not included end-users in their design. Ineffective role demarcations may result from limited consumer input during role introduction, thereby inhibiting end-user ability to make informed choices about NP/PP services. New Zealand’s Ministry of Health has emphasised co-design as integral to ensuring that health service delivery centres around the needs of individuals, rather than health care providers [2]. Consumers should be involved in designing and implementing advanced practitioner roles so that these roles align with individual expectations of patient-centred care and operate to their full value [46, 49]. This is particularly important given findings of this research emphasising the role confidence plays in facilitating satisfaction with services NPs/PPs provide; existing quality of care frameworks and their focus on patient care experience also reinforce these concepts [50–52].

New Zealand nursing research [53, 54] has shown that advanced practitioners operating in practice often cease working in patient-centred ways (despite this being key to the contexts participants highlighted in the present study) and instead take on roles as GP substitutes. In recommending consumer involvement, we recognise that individual preferences will change, particularly in those unfamiliar with service offerings [55]. We, therefore, suggest that co-design does not occur in a one-off tokenistic manner, but instead involve health professionals in continuous dialogue with those receiving their services. Such a system where practitioners work to their full potential could mean bringing aspects of tertiary-level services into primary health care and place GPs into diagnostic, complex comorbidity roles, itself potentially affecting retention and workforce burn-out [1], but also affecting how these health professionals are perceived by consumers wanting more patient-centred care delivery.

The present study speaks to consumer awareness of the passion advanced practitioners have in their expanded positions. This passion is important to cultivate in advanced practitioner candidates living in rural areas of workforce shortage [2] and in minority communities to mitigate future shortages, as supported by NP research [53, 54]. While there is some postgraduate funding at the national level to support NP training, there is no dedicated funding for PP candidates. Furthermore, postgraduate funding for both these pathways lags behind medicine, which received 63% of all funding in 2016/17 [56]. Targeted training funding tied to specific roles remains a viable way to support the continued introduction of these health professionals. It is paramount to capture this opportunity to further the introduction of NP and PP roles, as both nursing and pharmacy have capacity for expanding their health service contribution; in New Zealand pharmacy is one of the youngest health professions and nursing is the single biggest health profession.

The purpose of conducting a realist evaluation is to form theories transferable to other situations, not necessarily to have generalisability [34]. The present study included perspectives from individuals receiving services from two distinct provider groups across New Zealand. Consumer views are often underrepresented. In raising them separately from other stakeholders, this article provides the opportunity to consider the value of effectively engaging with end-users. Using a teacher–learner cycle, theories were able to be posed to research participants and an interchangeable learning process was able to ensue with information-rich participants [35]. Unlike Manzano's (2016) [35] suggestion that those using health services remain sensitised to the outcomes of a programme, this work recognises that in addition to a recognition of programme outcomes, participants remained knowledgeable of contexts underlying advanced practitioner practice. In clinical practice, this work, therefore, suggests a need for health professionals and those involved in health service planning to be more aware of what it is that consumers see and react to when receiving health services. Methodologically, this work cautiously hypothesises that as in any learning environment, teachers must be aware that learners may have more to offer than expected. Going beyond more recent work on realist interviewing [57], there remains a need for greater awareness of the experiential role consumers can play in theory consolidation through supporting the generation of linked dyads and context, mechanism, and outcome triads outside of their perceived realms of expertise. This is an important methodological consideration for realist researchers to carry forward.

Findings of this study need to be interpreted while considering potential limitations. These include the modest number of consumer participants and the fact that one interviewee participated in a much shorter interview than any other participant. Overall, study findings represent one stage of theory refinement, additional iterative alteration of final overarching theories developed as part of the wider study [33], will require testing as part of future research. Additional investigation is also required to determine whether patient-centred care as delivered by NPs/PPs occurs because of their position, the underlying training of nursing and pharmacy, practice pressures, or whether other providers can operate in this way when working in multidisciplinary teams. This work will become more relevant as NP and PP roles become more established and indeed may benefit from an exploration of preconceptions around the roles of health professions, including those of the more established medical profession. Additionally, there is room to examine more closely what patient-centred NP/PP care looks like for minorities and based on gender, and when delivered by nurses and pharmacists already established in their communities prior to entering advanced practice.

## Conclusion

This article contributes to a growing field of theory-driven advanced practitioner research. The highlighted contexts and mechanism emphasise a way of delivering health care that has potential to inform how NP/PP roles are introduced and managed, and how other service providers operate in primary health care. To safeguard continued consumer confidence and subsequent satisfaction in NPs/PPs, there is a need to ensure end-users feel known, are involved in their health care decision-making, and that providers maintain their passion in delivering services. Consequently, consumer involvement in discussions around health care service delivery is necessary. This is particularly important given the wider New Zealand and global context of ageing populations, increasing long-term comorbidities, and deficits in health workforce supply.

## Data Availability

Data sharing is not applicable to this article as no datasets were generated or analysed during the current study.

## Abbreviations

GP: General practitioner
NP: Nurse practitioner
PP: Pharmacist prescriber

## Footnotes

1 An initial theory, developed as part of the original literature review and refined throughout this article, is presented at the conclusion of the ‘Results’ section.
